# Cotranslational protein targeting to the membrane: Nascent-chain transfer in a quaternary complex formed at the translocon

**DOI:** 10.1038/s41598-018-28262-8

**Published:** 2018-07-02

**Authors:** Albena Draycheva, Sejeong Lee, Wolfgang Wintermeyer

**Affiliations:** 1 Department of Physical Biochemistry, Max Planck Institute for Biophysical Chemistry, 37077 Göttingen, Germany; 2 Present Address: Chemistry Research Laboratory, University of Oxford, OX1 3TA Oxford, UK

## Abstract

Membrane proteins in bacteria are cotranslationally inserted into the plasma membrane through the SecYEG translocon. Ribosomes exposing the signal-anchor sequence (SAS) of a membrane protein are targeted to the translocon by the signal recognition particle (SRP) pathway. SRP scans translating ribosomes and forms high-affinity targeting complexes with those exposing a SAS. Recognition of the SAS activates SRP for binding to its receptor, FtsY, which, in turn, is primed for SRP binding by complex formation with SecYEG, resulting in a quaternary targeting complex. Here we examine the effect of SecYEG docking to ribosome-nascent-chain complexes (RNCs) on SRP binding and SAS transfer, using SecYEG embedded in phospholipid-containing nanodiscs and monitoring FRET between fluorescence-labeled constituents of the targeting complex. SecYEG–FtsY binding to RNC–SRP complexes lowers the affinity of SRP to both ribosome and FtsY, indicating a general weakening of the complex due to partial binding competition near the ribosomal peptide exit. The rearrangement of the quaternary targeting complex to the pre-transfer complex requires an at least partially exposed SAS. The presence of SecYEG-bound FtsY and the length of the nascent chain strongly influence nascent-chain transfer from SRP to the translocon and repositioning of SRP in the post-transfer complex.

## Introduction

Extracytosolic proteins constitute about one third of the bacterial proteome and there are various pathways to target these proteins to the membrane in order to insert them into the lipid bilayer or translocate them through the membrane. In gram-negative bacteria, most inner-membrane proteins are cotranslationally targeted to the SecYEG translocon via the signal recognition particle (SRP) pathway. SRP rapidly scans ribosomes by forming transient complexes. On binding to a ribosome-nascent-chain complex (RNC) exposing the signal-anchor sequence (SAS) of a membrane protein, SRP undergoes a conformational change that results in the formation of a high-affinity complex^1–6^. The formation of the complex of SRP and RNC protects the SAS from other interaction partners such as ribosome-associated chaperones and nascent-chain modifying enzymes^7–10^. Additionally, binding to RNCs activates SRP by engaging the C-terminal M domain of SRP protein Ffh in SAS binding and the N domain in interaction with ribosomal protein uL23. In this way the NG (GTPase) domain becomes available for binding to the homologous NG domain of the SRP receptor, FtsY^11–15^. Similarly, the free form of FtsY is in an inactive”closed” state where the N-terminal, membrane-binding A domain and the C-terminal NG domain are engaged in a strong interaction with one another^16^. On binding to the translocon in the membrane, FtsY is activated by an engagement of the A domain with SecYEG and membrane phospholipids^16,17^. This frees the NG domain of FtsY for the interaction with the homologous NG domain of SRP protein Ffh^18^.

These activation events lead to an increased affinity of SRP binding to FtsY and take place at the ribosome or at SecYEG^1,2,16,19,20^. In addition, the interaction between the SecYEG translocon and the ribosome is enhanced in the presence of the nascent chain of a membrane protein^21,22^. The increased affinity between SRP and FtsY and between RNC and SecYEG favors the assembly of the quaternary targeting/transfer complex at the translocon^21,22^. However, a detailed description of the events taking place at the membrane is still lacking. Structural and biochemical data suggested that SRP and SecYEG interact with the ribosome in a mutually exclusive manner^20,23^. Furthermore, it was proposed that FtsY and the ribosome compete for binding to the C5 loop of SecY. Biochemical studies revealed a rearrangement of FtsY on SecYEG which allows docking of the ribosome to form a ternary complex^24^. The interplay between SecYEG and SRP during RNC transfer and of SAS handover to SecYEG is not understood in detail. Similarly, the mechanism by which the activated targeting complex rearranges to the transfer complex remains undefined.

We set out to investigate the formation of the quaternary complex of components from *Escherichia coli* during RNC targeting and SAS transfer to SecYEG at the membrane. We performed equilibrium titrations monitored by fluorescence resonance energy transfer (FRET) between two fluorophores to quantify the interaction of SRP bound to translating ribosomes in different functional states with FtsY bound to SecYEG embedded in phospholipid-containing nanodiscs. We also determined changes of the affinity of the SRP–FtsY complex in the course of targeting and transfer of the RNC and the handover of the SAS from SRP to SecYEG applying FRET and proteinase protection assays.

## Results

### Concurrent binding of SRP and SecYEG to the ribosome

We first addressed the question whether SRP and the SecYEG translocon can bind concurrently in a simplified system lacking the SRP receptor FtsY. We studied the formation of a ternary targeting complex on stalled RNCs carrying nascent chains of leader peptidase (Lep) of 50 or 75 amino acids in length (Material and Methods)^2^. As the peptide exit tunnel covers 35 amino acids of Lep^3^, these RNCs expose 15 (Lep50) or 40 (Lep75) N-terminal amino acids, i.e., about half or all of the SAS of Lep^25^. Fluorescence-labeled Lep-RNCs carried a Bodipy FL (Bpy) fluorophore at the N-terminal methionine of the nascent chain. As the SRP receptor FtsY does not bind to the detergent-solubilized translocon^24^, the SecYEG translocon was inserted into nanodiscs containing a mixture of phospholipids characteristic for the plasma membrane of *E*. *coli*^16,24,26^; the preparation yields nanodiscs containing a single copy of SecYEG, as verified by quantitative mass spectrometry (Material and Methods), that was functionally competent^16,21^.

**Figure 1:**
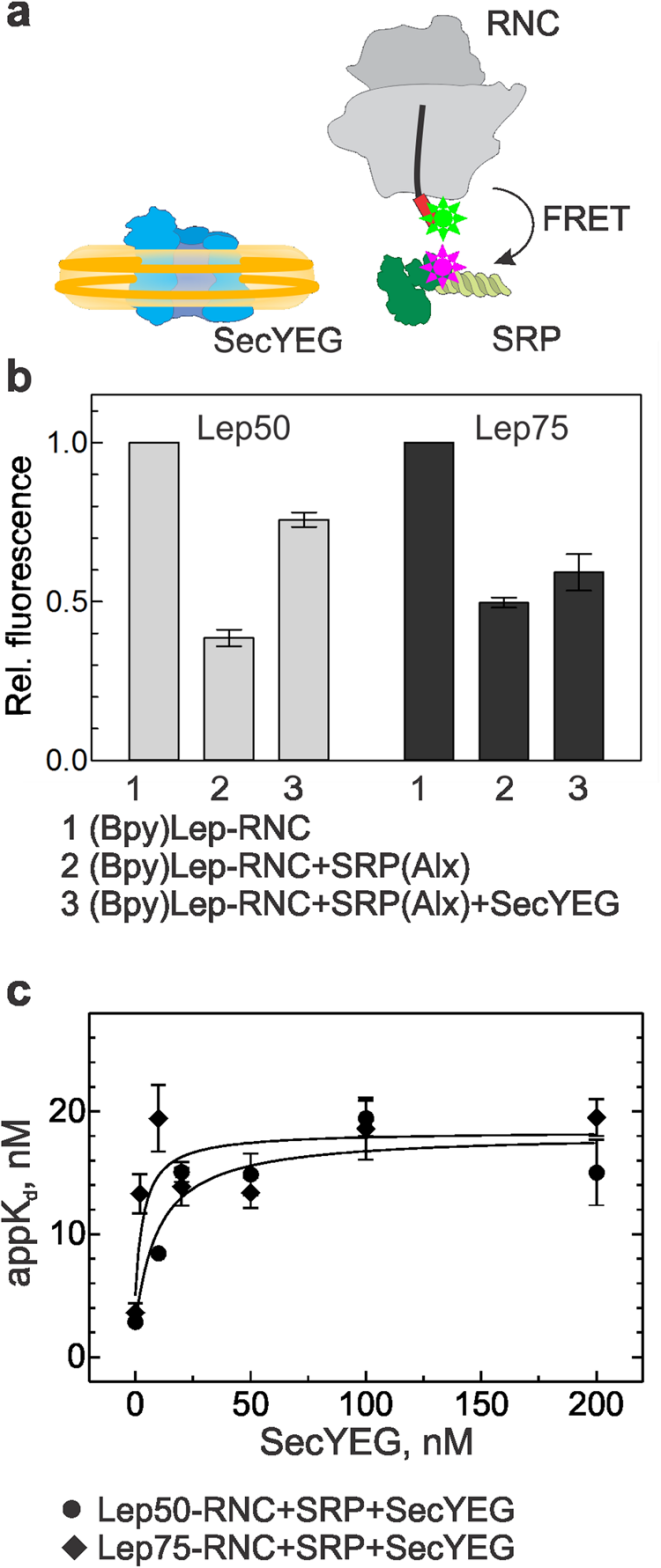
Concurrent binding of SRP and SecYEG to Lep-RNCs. (**a**) Schematic of the interaction partners studied by FRET between Bpy at the N terminus of the nascent SAS (red box) emerging from the ribosome and Alx attached to the M domain of SRP protein Ffh (labels indicated by stars). SecYEG (blue) is inserted into nanodiscs (yellow). (**b**) Successive binding of SRP(Alx) (100 nM) and SecYEG (400 nM) to (Bpy)Lep50-RNC (10 nM) or (Bpy)Lep75-RNC (10 nM) monitored by FRET. Bpy fluorescence is plotted relative to the initial signal set to 1.0. Error margins represent SEM, n = 2–4. The FRET values observed with Lep50 and Lep75 in the presence of SecYEG (columns 3) are significantly different (p < 0.015, according to an unpaired two-tailed t-test) (**c**) Effect of SecYEG binding on the affinity of SRP–Lep-RNC complexes. Apparent K_d_ values of SRP binding to Lep50-RNC or Lep75-RNC were measured by titrations in the presence of increasing concentrations of SecYEG (Supplementary Fig. S1a,b). Data were analyzed by fitting equation (2) (Material and Methods). Error margins represent SEM, n = 2–3.

To examine concurrent binding of SRP and SecYEG to RNCs, we measured the effect of the presence of SecYEG on SRP binding to Lep50- and Lep75-RNC. We monitored complex formation by FRET between Bpy at the N terminus of the nascent peptide as donor^27–29^ and Alexa555 (Alx) at position 430 in the M domain of SRP-protein Ffh as acceptor (Fig. 1a). These labels do not interfere with complex formation^2,3,7^ and directly monitor the interaction of the labeled nascent chain with the labeled M domain of Ffh. SRP(Alx) binding to (Bpy)Lep50- or (Bpy)Lep75-RNCs in the absence of SecYEG decreased the donor fluorescence due to FRET to about 40% or 50%, respectively, relative to the initial signal (Fig. 1b). The addition of SecYEG in saturating amount (cf. Fig. 1c) partially reversed the FRET effect on the Lep50-RNC–SRP complex to 80% of the initial signal. The reversal of FRET was somewhat smaller on the Lep75-RNC–SRP complex, indicating a different arrangement of the FRET label(s) in the complexes with shorter and longer nascent chains. The fact that some FRET persisted after the addition of SecYEG suggests that SRP was not displaced from the ribosome by SecYEG but remained bound to either RNC, presumably in a different arrangement, as indicated by the different FRET levels observed in the absence and presence of SecYEG.

To characterize the binding of SRP and SecYEG quantitatively, we performed equilibrium titrations, using the same FRET pair to monitor SRP(Alx) binding to (Bpy)Lep50-RNC or (Bpy)Lep75-RNC in the absence or presence of increasing amounts of SecYEG (Supplementary Fig. S1a,b). The apparent K_d_ (appK_d_) of SRP binding to Lep50- or Lep75-RNC increased from 3–4 nM observed in the absence of SecYEG to 15–20 nM in the presence of saturating amounts of SecYEG (Fig. 1c). Such behavior is indicative of concurrent, partially competitive binding of SRP and SecYEG to the RNCs, forming a ternary complex, where the appK_d_ of SRP binding was increased by a factor α = 5 ± 2 (equation (2), Material and Methods).

### Formation of the quaternary transfer complex

**Figure 2:**
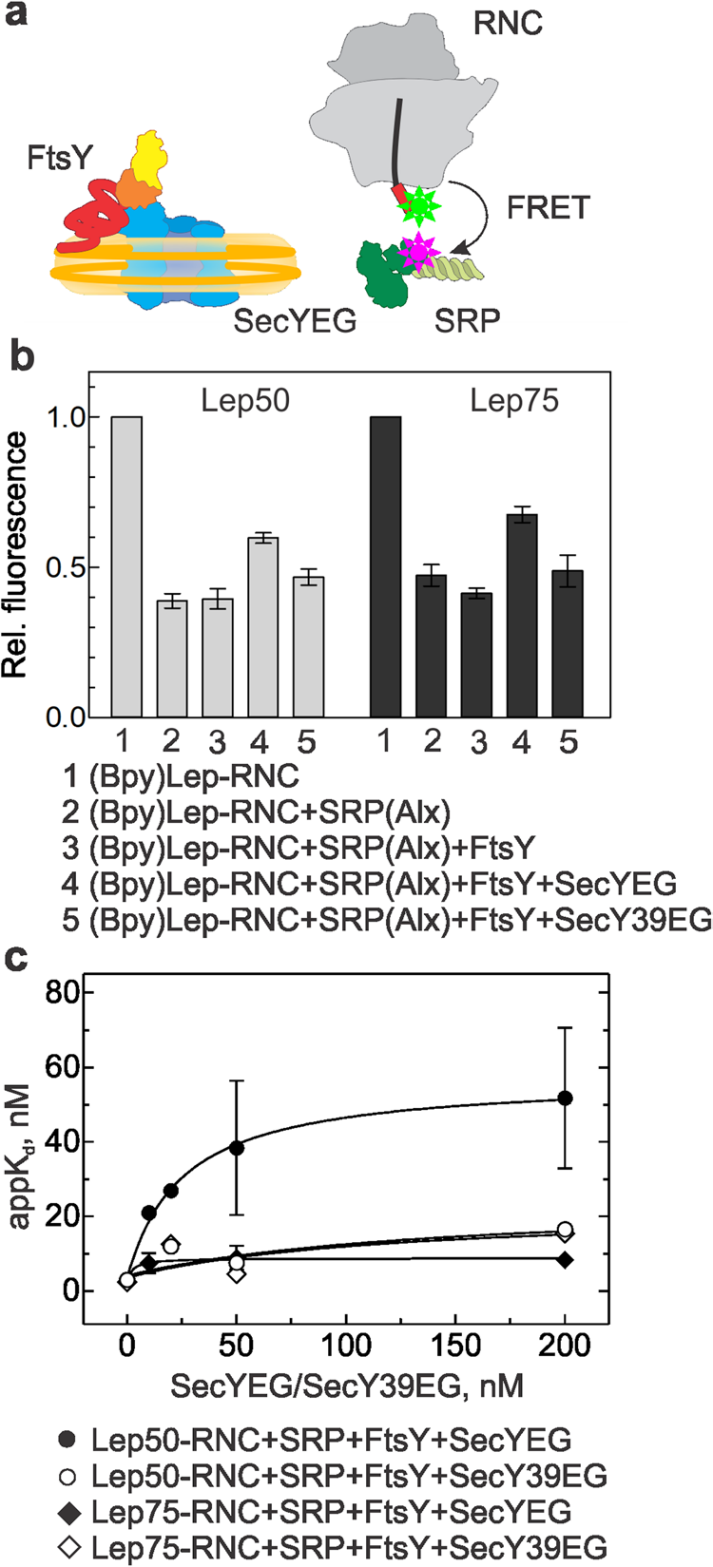
SRP binding to Lep-RNCs in the presence of SecYEG–FtsY. (**a**) Schematic of the interaction partners studied by FRET. The NG domain of FtsY in indicated in orange and yellow, the unstructured A domain in arbitrary conformation (red). (**b**) Binding of SRP(Alx) to (Bpy)Lep50-RNC or (Bpy)Lep75-RNC. Concentrations as in Fig. 1. The fluorescence of (Bpy)Lep-RNC was measured before and after the successive addition of SRP(Alx), SecYEG–FtsY, or SecY39EG–FtsY. Bpy fluorescence is plotted relative to the initial signal set to 1.0. Error margins represent SEM, n = 2–4. (**c**) Effect of SecYEG–FtsY binding on the affinity of SRP–Lep-RNC complexes. Apparent K_d_ values for SRP binding to Lep50-RNC or Lep75-RNC were measured in the presence of FtsY and increasing concentrations of SecYEG (Supplementary Fig. S1c–f). Data were fitted to equation (2) (Material and Methods). Error margins represent SEM, n = 2–4.

We performed analogous experiments including the SRP receptor, FtsY, which is essential for SRP–RNC targeting to the translocon and the release of SRP^30,31^. Again we used FRET to monitor the binding of SRP to the SAS in the presence of the other binding partners (Fig. 2a). On binding of SRP(Alx) to (Bpy)Lep50-RNC or (Bpy)Lep75-RNC, the Bpy donor signal decreased to about 40–50% (Fig. 2b), as above. The addition of a saturating amount of SecYEG in complex with FtsY only partially restored the donor signal (60% of the initial signal for (Bpy)Lep50-RNC and 70% for (Bpy)Lep75-RNC (Fig. 2b)), indicating that SRP(Alx) was retained in the complex. The addition of FtsY alone did not cause a FRET change, which indicates that the effect is due to the interaction of SecYEG with the RNC–SRP complex, The decrease of FRET between labels at position 430 in the M domain of SRP protein Ffh and at the N terminus of the nascent chain indicates a rearrangement of the complex during which the labels move apart. We observed a similar effect when the non-fluorescent FRET acceptor QSY9 was attached to position 84 in the N domain of Ffh, which is not in contact with the SAS (Supplementary Fig. S2). This suggests that, upon addition of SecYEG or the SecYEG–FtsY complex, the SAS moves away from SRP, presumably establishing an interaction with SecYEG (see below).

To examine the effects of SecYEG binding to the nascent chain and the ribosome separately, we have used a SecY mutant, SecY39, which has a single amino acid exchange, R357H, in cytosolic loop 5 and is deficient in SRP-dependent membrane integration of inner-membrane proteins *in vivo*^32,33^. FRET titrations using Alx-labeled SecY39EG embedded in nanodiscs revealed that the mutation abrogates SecYEG binding to vacant ribosomes (Supplementary Fig. S3a), as the related R357E mutation^34^, whereas binding to Lep-RNCs was affected only slightly (Supplementary Fig. S3b). The addition of SecY39EG–FtsY to the RNC–SRP complex had no effect in reversing the SRP effect on donor fluorescence (Fig. 2b), indicating that the effect seen with non-mutated SecYEG was due to ribosome binding.

Taken together these results suggest that in the quaternary complex formed with SRP, FtsY, SecYEG and Lep50- or Lep75-RNC the efficiency of FRET between the labels on the SAS and on SRP was low, compared to the binary RNC–SRP complex. This indicates a reorientation of SRP on the ribosome upon binding of SecYEG or SecYEG–FtsY. To quantify the effect on the binding affinity, we titrated (Bpy)Lep50- or (Bpy)Lep75-RNC in complex with FtsY–SecYEG with SRP(Alx) (Supplementary Fig. S1c,d). The appK_d_ of SRP binding to Lep50-RNC increased to 50 ± 20 nM (α ≈ 20; equation (2), Material and Methods), and the effect was smaller with Lep75-RNC (α ≈ 4) (Fig. 2c).

To dissect the thermodynamic contributions of ribosome and nascent-chain binding on quaternary complex formation, we tested the influence of the SecYEG-ribosome interaction on SRP–Lep-RNC complex formation with the SecY39EG mutant that is impaired in ribosome binding. The addition of SecY39EG–FtsY had a rather small effect (up to about five-fold change; Supplementary Fig. S1e,f) on the apparent affinity of SRP binding to Lep50- or Lep75-RNC (Fig. 2c). This suggests that the increase of the appK_d_ of SRP binding is due to the interaction of the translocon with the ribosome, implying partially overlapping binding sites on the ribosome. In control experiments we measured the apparent affinity of SRP binding to vacant ribosomes and observed no change upon addition of SecYEG–FtsY (Supplementary Fig. S4a). Furthermore, an RNC carrying the nascent chain of outer-membrane protein A (proOmpA75-RNC), which lacks a SAS, interacted with SecYEG with K_d_ = 15 ± 3 nM, as Lep-RNC (Supplementary Fig. S4b). Furthermore, we observed no affinity change of SRP binding to proOmpA-RNC upon addition of SecYEG–FtsY (Supplementary Fig. S4c). At the same time, however, we observed a strong decrease of the FRET amplitude upon binding of SecYEG–FtsY (Supplementary Fig. S4c), which is in keeping with a reorientation of SRP upon formation of the quaternary complex.

### Targeting complex formation and SAS transfer lower the SRP–FtsY affinity

**Figure 3:**
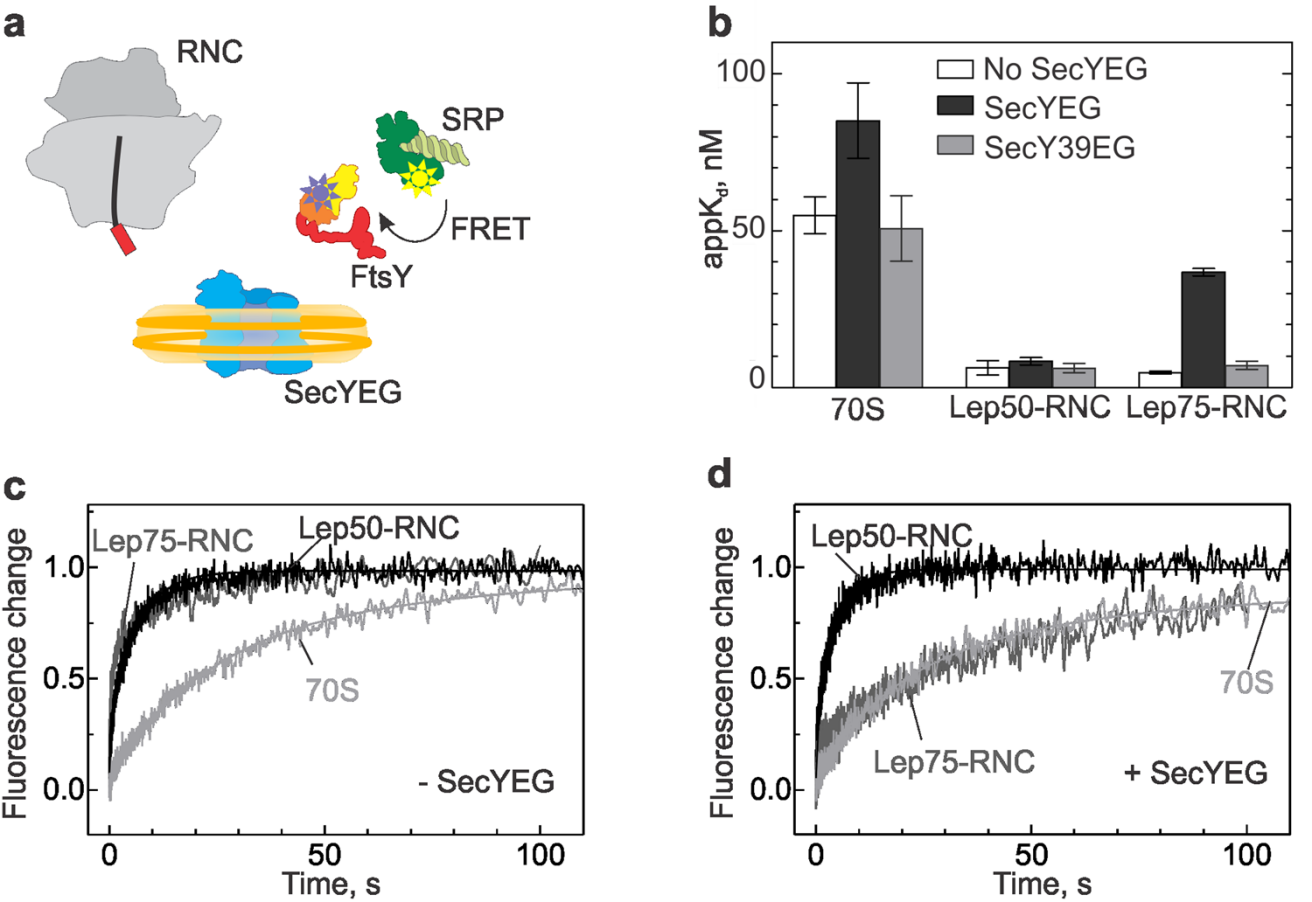
Effect of SecYEG on the SRP–FtsY interaction on ribosomes or Lep-RNCs. (**a**) Schematic of the components used for studying the SRP–FtsY interaction in the quaternary complex. (**b**) The affinity of the SRP interaction with FtsY was measured in complex with vacant ribosomes (70S) and Lep-RNCs with nascent chains of different length (50 or 75 amino acids), upon addition of SecYEG or SecY39EG in nanodiscs. The addition of empty nanodiscs had no effect (data not shown). Apparent K_d_ values are presented as mean ± SEM (n = 2–3). (**c**,**d**) Time courses of FtsY binding to SRP in the presence of 70S ribosomes/RNCs (**c**), or 70S ribosomes/RNCs and SecYEG (**d**) were monitored by FRET between FtsY(Bpy) and SRP(MDCC). Acceptor fluorescence was followed after rapid mixing of FtsY(Bpy) with SRP(MDCC) in complex with 70S, Lep50-RNC, or Lep75-RNC. The results of double-exponential fits are indicated by continuous lines.

Next we asked the question whether the altered interaction between SRP and the RNC that is caused by the binding of SecYEG has an influence on the interaction of SRP and FtsY. We performed equilibrium titrations, monitoring FRET between labels in SRP (MDCC at position 235 in the G domain of Ffh) and FtsY (Bpy at position 487 in the N domain) (Fig. 3a); these labels were validated earlier^35^. The resulting apparent K_d_ values are summarized in Fig. 3b. SecYEG and FtsY formed a rather strong complex, appK_d_ = 200 nM^24^, and the binding of SecYEG strengthened the interaction of FtsY with SRP (appK_d_ decreasing from ~ 500 nM to about 50 nM;^2,16^). Similarly, the interaction with FtsY was enhanced when SRP was bound to non-translating ribosomes (appK_d_ = 50 nM;^1,18^). The addition of SecYEG or SecY39EG had very little or no further effect. Binding of SRP to Lep50-RNC enhanced the affinity of SRP to FtsY (appK_d_ = 5–10 nM), whereas SecYEG or SecY39EG had no effect on the SRP–FtsY interaction. By contrast, with RNCs carrying a longer nascent chain (Lep75), the presence of SecYEG strongly reduced the apparent affinity of FtsY binding to RNC-bound SRP (Supplementary Fig. S5a), increasing the appK_d_ about ten-fold (to 40 ± 10 nM), whereas mutant SecY39EG had no such effect (Fig. 3a; Supplementary Fig. S5b). The increase of the appK_d_ was hyperbolic and saturated at a SecYEG concentration of 0.6–1 µM (Supplementary Fig. S5c). This reflects the FtsY–SecYEG interaction upon formation of a quaternary complex in which the SRP–FtsY interaction is weakened. Although mutant SecY39EG has no quantitative effect on the interaction between SRP and FtsY on Lep75-RNCs, it induces a rearrangement of the complex, as indicated by changes of FRET between labels in SRP and FtsY (Supplementary Fig. S5b).

The stabilizing effect of the nascent chain on the interaction between RNC-bound SRP and FtsY is mainly due to an accelerated rearrangement following initial binding^2^. To examine whether the destabilizing effect of SecYEG may be related to competition between SRP and SecYEG for binding to the nascent chain, we compared the effect of SecYEG on time courses of association of FtsY and SRP bound to non-translating ribosomes, Lep50-RNC, or Lep75-RNC (Fig. 3c,d). We monitored complex formation by changes of FRET between labels in Ffh and FtsY, as above, measuring the increase in acceptor (Bpy) fluorescence. FtsY binds more rapidly to SRP when it is bound to Lep50-RNC (average half-life time around 1 s), rather than to non-translating ribosomes (half-life time around 15 s), as observed previously^2^, and similar kinetics we observed with Lep75-RNC (Fig. 3c). Notably, upon addition of SecYEG the rate of FtsY binding to the Lep75-RNC–SRP complex was lowered to the rate observed with non-translating ribosomes, whereas the time course of FtsY binding to SRP on Lep50-RNC did not change (Fig. 3d). This behavior strongly suggests that the nascent peptide of Lep75-RNC, which is long enough to be inserted into SecYEG (see below), by dissociation from the M domain of Ffh/SRP causes a rearrangement of SRP that slows down complex formation with FtsY, resulting in a ten-fold increase of the appK_d_.

### Nascent-chain transfer in the quaternary complex

**Figure 4:**
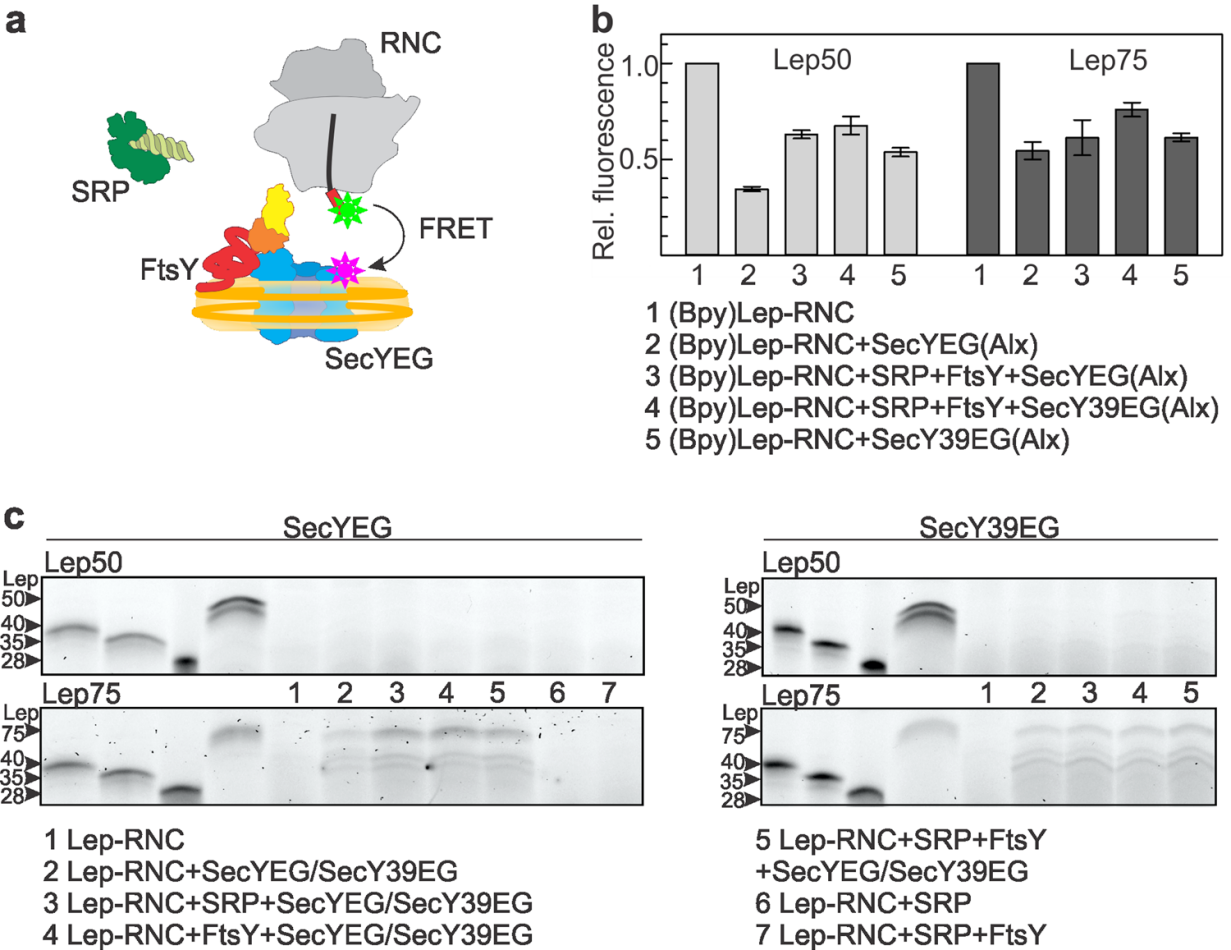
Nascent-chain transfer and insertion into SecYEG in the quaternary complex. (**a**) Schematic of the interaction partners and label positions used for studying the interaction between nascent SAS and the translocon in the quaternary complex. (**b**) Nascent-chain transfer monitored by FRET between (Bpy)Lep-RNC and SecY labeled with the acceptor (Alx) in the SecYEG complex. The interaction of (Bpy)Lep50-RNC or (Bpy)Lep75-RNC and SecYEG(Alx) was monitored by FRET with different complexes, as indicated. Concentrations as in Fig. 1. Donor fluorescence is plotted relative to the initial value measured with the respective (Bpy)Lep-RNC prior to any addition, set to 1.0. Error margins indicate SEM (n = 2–4). (**c**) Proteinase accessibility of Bpy-labeled Lep50 or Lep75 nascent chains in complexes with SecYEG or SecY39EG and SRP/FtsY. Nascent-peptide accessibility was monitored by proteinase K (PK) digestion followed by RNaseA digestion and separation of Lep peptides by denaturing gel electrophoresis. Lep peptide standards (28, 35, 40, and 50, or 75 amino acids in length (four leftmost lanes) were prepared by RNaseA digestion of the respective Lep-RNCs without PK treatment. N-terminal peptides are visualized by the fluorescence of Bpy attached to the N termini of the nascent chains. Uncropped gels with the complete sample lanes are presented in Supplementary Fig. S6.

In the presence of SecYEG the efficiency of FRET between labels in SRP and the nascent chain decreased (Fig. 2b), along with a lowered affinity of SRP binding to the Lep50-RNC (Fig. 2c). After extension of the nascent chain to 75 amino acids the complex of SRP and FtsY also assumed a lower-affinity state (Fig. 3b). This raises the question whether these events are connected to the transfer of the SAS to SecYEG. We examined the interaction of the SAS with SecY, monitoring FRET between the Bpy donor at the N terminus of the nascent peptide and the acceptor Alexa555 (Alx) attached to an engineered Cys at position 179 in the cytosolic C3 loop of SecY (Fig. 4a). The label did not affect the binding of SecYEG to Lep-RNCs (Supplementary Fig. S3b). With Lep50-RNC, the donor fluorescence decreased to 35% of the initial signal, whereas with Lep75-RNC the fluorescence signal decreased to only 54% (Fig. 4b). In the quaternary complex the donor fluorescence decreased to a similar level, 60%, with both Lep50- and Lep75-RNCs. This indicates that the SAS and SecYEG are in close proximity and yet the complex differs from the complex without SRP and FtsY, in particular the complex with Lep50-RNC (Fig. 4b). Experiments with SecY39EG, which has little effect on the SRP-RNC interaction, showed less FRET for Lep50-RNC (donor fluorescence decrease to 70%), compared to non-mutated SecYEG. No appreciable FRET differences were observed in the SecYEG complexes with Lep50- or Lep75-RNC in the absence or presence of SRP and FtsY, or with either SecY39EG or SecYEG (Fig. 4b). These results suggest that a quaternary complex can be formed, despite the loss of interaction between the ribosome and SecY39EG. However, this does not clarify whether the close approach between the SAS and SecYEG leads to nascent-chain transfer to SecYEG and membrane insertion.

To analyze at which stage of the quaternary complex assembly the SAS is transferred to SecYEG we performed proteinase K digestion experiments on Lep-RNCs in different complexes^21^. Proteinase K efficiently cleaves the nascent chain in RNCs in the absence of SecYEG or in the complex with SRP, or SRP and FtsY. Lep50 is N-terminally processed by the proteinase even in the presence of SecYEG (Fig. 4c), indicating that the exposed part of the nascent chain, i.e., the 15 N-terminal amino acids, is too short to insert into the translocon and be protected. By contrast, Lep75 is protected by both SecYEG and SecY39EG (Fig. 4c), independent of the presence of SRP and FtsY, as observed previously^21^. It appears that the extra 25 amino acids of Lep75-RNC enable the SAS to be inserted into the translocon (or even to enter the phospholipids surrounding the translocon in the nanodiscs). The length of the protected peptide is up to 75 amino acids, although somewhat shorter peptides (around 35–40 amino acids in length), which probably result from cleavage at additional unprotected sites, are also formed. These results suggest that the growing nascent chain can interact with SecYEG, but that the stable insertion requires more than 15 amino acids exposed outside the exit tunnel.

### Rearrangement of SRP and FtsY in the post-transfer complex

**Figure 5:**
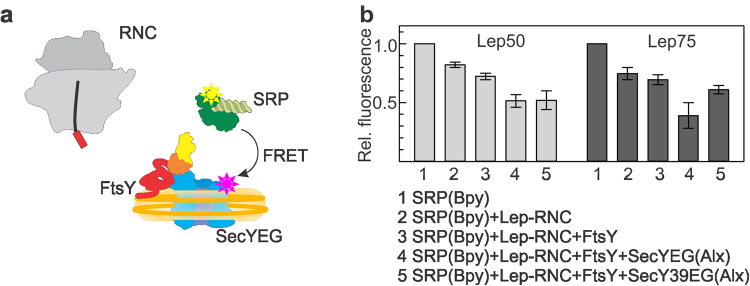
Rearrangement of SRP in the post-transfer complex. (**a**) Schematic of the components and label positions used for monitoring the SRP rearrangement. (**b**) Fluorescence or FRET with labeled SRP and SecYEG were measured in complexes with Lep50-RNC or Lep75-RNC. SRP was labeled with the Bpy donor and SecY with the Alx acceptor. Fluorescence (compare 1, 2, and 3) or FRET (compare 4 and 5) were monitored by the fluorescence of SRP(Bpy) and are plotted relative to the initial fluorescence of SRP(Bpy) alone, set to 1.0. Error margins indicate SEM (n = 2–4).

Our observations suggest a model in which SRP and FtsY are part of the post-transfer complex after rearranging from their initial positions. In order to check how SAS transfer reorganized the quaternary complex we monitored the arrangement of SRP relative to the C3 loop of SecY. We measured FRET between SRP labeled with Bpy at position 430 (SRP(Bpy))^2^ and SecY labeled with Alexa555 (SecYEG(Alx)) in the C3 loop (Fig. 5a). In the complex with Lep50-RNC we observed a decrease of donor fluorescence (to 50% of the initial signal) upon binding of SecYEG, and a larger effect (to about 35%) with Lep75-RNC (Fig. 5b). This indicates that, upon nascent-chain transfer, the SRP/Ffh M domain, which carries the label, moves closer to SecY rather than being displaced from the complex. Presumably SRP in complex with FtsY vacated the C5 loop of SecY, where protein uL23 interacts, and relocated to the C3 loop. This rearrangement probably takes place after SAS transfer to SecYEG, which lowers the affinity of the interaction between SRP and FtsY (Fig. 3b). The efficiency of FRET between SRP and SecYEG in the quaternary complex with Lep75-RNC decreased when mutant SecYEG was used (Fig. 5b, compare columns 4 and 5). This indicates that the interaction of SecY with the ribosome, presumably at protein uL23, has an important function in maintaining the steric arrangement in the complex. This is in line with the observation (Fig. 2b) that the addition of FtsY–SecY39EG did not decrease the efficiency of FRET between Lep-RNC and SRP in the quaternary complex and did not affect the affinity of SRP binding to FtsY (Fig. 3b).

## Discussion

**Figure 6:**
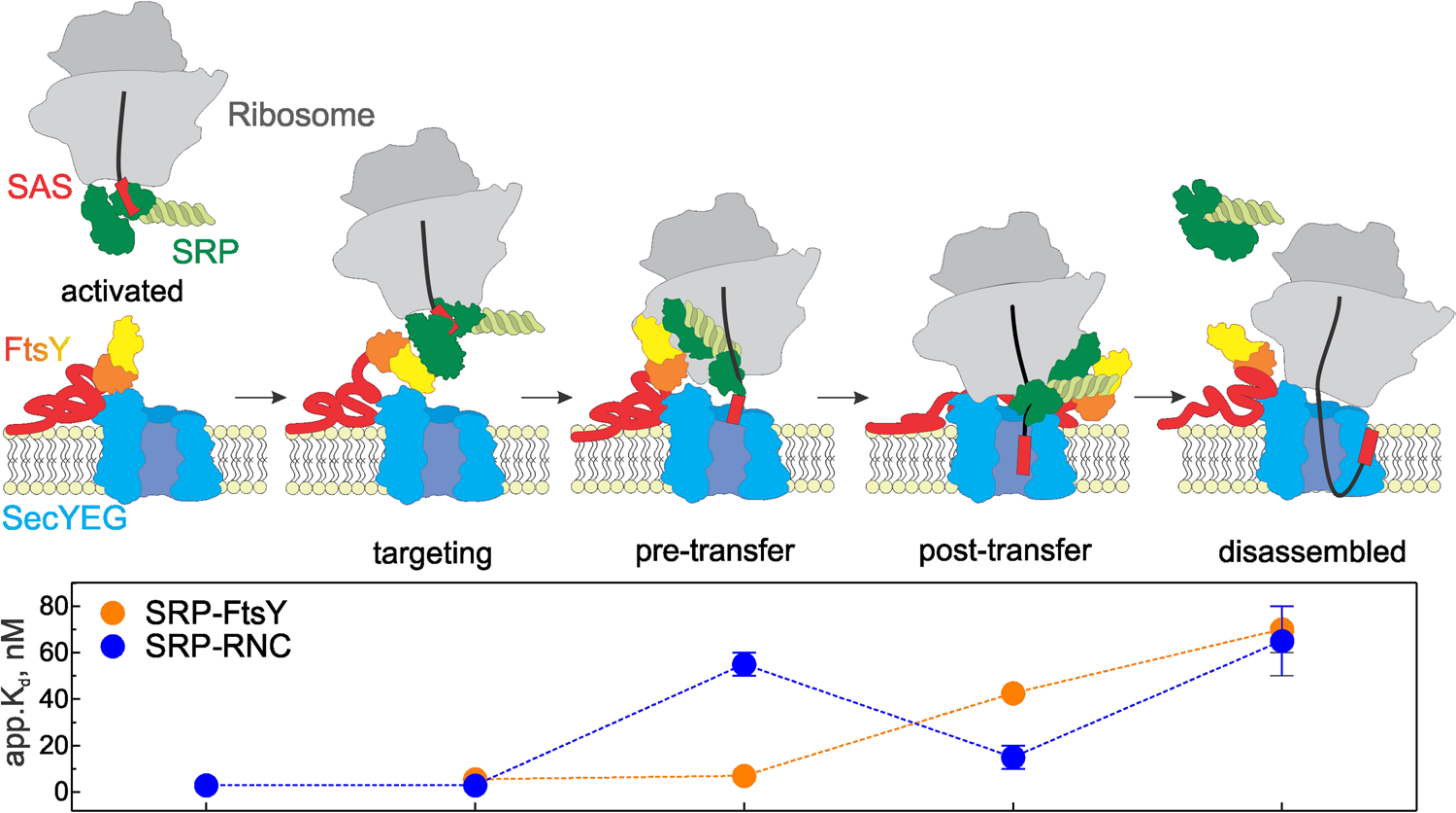
Schematic of RNC targeting and nascent-chain transfer to SecYEG in a quaternary membrane-associated complex. In the activation phase, an RNC exposing the SAS of an inner-membrane protein forms a high-affinity complex with SRP that leads to activation of SRP for an efficient interaction with the SRP receptor FtsY via the exposed Ffh NG domain (green) and the homologous NG domain (yellow/orange) of FtsY which is exposed upon binding of the unstructured A domain to SecYEG^14,16^. Targeting complex formation prepares SRP for the transfer of the SAS to SecYEG in that SRP rearranges on the ribosome and vacates the binding site of SecYEG near the peptide exit. This results in the pre-transfer complex in which the SRP-RNC interaction is destabilized, but that of SRP─FtsY remains tight (lower panel). Elongation of the nascent chain to an insertion-competent length triggers the transfer of the SAS to SecYEG to form the post-transfer complex. After the transfer, the complex rearranges and the SRP-FtsY interaction becomes weaker. Following GTP hydrolysis on both Ffh/SRP and FtsY, the post-transfer complex is disassembled and the targeting machinery can associate with another RNC, or the same ribosome exposing an upstream TM segment, to enter a new round of targeting. Apparent Kd values for the disassembled state are for the complexes of SRP with ribosomes, FtsY with SRP bound to ribosomes^1,18^, or SRP with FtsY bound to SecYEG^16^.

In this work we investigated mechanisms of cotranslational targeting and transfer of nascent inner-membrane proteins to the SecYEG translocon. We examined how the initial quaternary targeting complex rearranges to a pre-transfer complex which consists of an RNC with bound SRP and the SecYEG translocon with bound FtsY and upon transfer of the SAS to a post-transfer complex (Fig. 6). The transitions involve discrete conformational changes, as indicated by changes of FRET between various constituents of the complex, which lower the affinity of interactions and result in a quaternary post-transfer complex. The transitions entail the concerted rearrangement of the NG domains of SRP and FtsY, in that SRP vacates ribosomal protein uL23 (this work) and FtsY clears the C5 loop of SecY^24,36^, such that SecYEG can dock onto the ribosome. These events previously were suggested to involve binding competition, in that the ribosome displaces FtsY from the complex with SecYEG which, in turn, competes with SRP for ribosome binding^20,23,37,38^. However, FtsY remains bound to SecYEG with unchanged affinity^16,24^. The present FRET measurements and equilibrium titrations reveal that, upon RNC binding to the SecYEG–FtsY complex, SRP does not dissociate from the RNC, but rather undergoes a conformational change that prepares SRP for SAS handover to the translocon. This is in accordance with structural work on the quaternary complex^37^, showing the ‘activated’ SRP–FtsY complex repositioned on the ribosome and the signal sequence extended towards the SecYEG translocon, beyond the hydrophobic binding groove of the SRP M domain. However, in that structural model SecYEG is positioned away from the ribosomal exit tunnel and the emerging nascent chain. The unusual position of SecYEG and the lack of interaction with the hydrophobic nascent chain may be due to the detergent used for complex preparation, rather than phospholipids, which are essential for signal sequence insertion and the functional interaction of FtsY and SecYEG^24^.

The exposure of the SAS of an inner-membrane protein on the ribosome outside of the peptide exit tunnel strongly increases the affinity of SRP binding to the ribosome and induces rearrangements between the NG and M domains of SRP protein Ffh^1,18,39^. The binding of the SAS to the hydrophobic cleft of the M domain is accompanied by an intradomain rearrangement and a movement of the NG domain of Ffh which increases its accessibility for the interaction with the NG domain of FtsY^18^. The finger loop in the M domain contributes to the binding of hydrophobic nascent chain segments and was implicated in SAS transfer to the translocon^35^. The binding of SecYEG (Fig. 1), and especially the SecYEG–FtsY complex (Fig. 2), diminishes the affinity of SRP to the RNC, but does not exclude SRP entirely. The effect depends on the presence of a SAS and a certain minimum length of the nascent chain exposed outside the peptide exit tunnel. When about 15 amino acids of the SAS are exposed (Lep50), FRET between labels at the N terminus of the nascent chain and the M domain of SRP is more efficient than when 40 amino acids are exposed (Lep75) (Fig. 1b, compare columns 2). This may suggest a closer approach of the N-terminal label of the shorter nascent chain to the label in the M domain of Ffh. The destabilization of the SRP–RNC complex by the binding of SecYEG–FtsY is particularly strong (20-fold) with the shorter nascent chain (Lep50), and also depends on the interaction of SecYEG with the ribosome (Fig. 2). This suggests that already in the quaternary complex with Lep50-RNC SRP is repositioned on the ribosome and has left its binding site on protein uL23 where SecY interacts. Despite the reduced affinity of the SRP–RNC complex, the affinity of the FtsY-SRP interaction remains high and stabilizes SRP in the quaternary complex (Fig. 3b). This is in accordance with structural data suggesting that the NG domain of SRP is detached from protein uL23 when it forms a complex with the homologous NG domain of FtsY^40^. Presumably, FtsY reverses conformational rearrangements induced in Ffh by SAS binding, thereby triggering the release of the SAS from the M domain and the transfer to SecYEG^18^. The strong interaction of SecYEG with the ribosome is another factor that contributes to the decrease in affinity between SRP and the RNC (Fig. 2c). When the interaction of SecYEG with the ribosome is impaired, as with the mutant SecY39EG, the affinity of the SRP–RNC complex is reduced about five-fold. On the other hand, SecYEG has no effect on the affinity of SRP binding to an RNC when the nascent chain lacks a SAS, as proOmpA75-RNC, but rather induces a conformational change in SRP, allowing SecYEG to interact with protein uL23 (Supplementary Fig. S4).

The switch of the targeting complex to the pre-transfer complex (Fig. 6) requires a partially or fully exposed SAS which provides specific interactions of SRP with the RNC. During the switch, SRP rearranges to release the nascent chain and to vacate uL23. The FRET data indicate that in the quaternary complex formed with Lep50-RNC the nascent chain is close to, and may reach into, the central pore of SecYEG (Fig. 4a), but is too short for stable insertion (Fig. 4c). Thus, the short nascent chain may transiently switch between SRP and SecYEG. As the nascent chain is growing, it becomes competent for insertion, as Lep75 is stably inserted into SecYEG and protected against proteinase K digestion. These results are consistent with data obtained with a eukaryotic translocon which indicated stable insertion when the nascent chain was extended to a length of 69 amino acids^41^. At that length the nascent chain apparently can reach into the translocation pore of SecY and, depending on the hydrophobicity of the SAS, may enter the pathways for either translocation into the periplasm or insertion into the membrane^42–44^.

In our *in-vitro* setup we used a non-hydrolyzable GTP analog, GDPNP, to prevent premature disassembly of the SRP–FtsY complex and multiple rounds of targeting. The fact that SAS transfer takes place in the presence of GDPNP indicates that the handover is independent of GTP hydrolysis. Rather the length of the nascent chain dictates the timing of the transfer. In the post-transfer complex we observed that, following conformational rearrangements, SRP is still in complex with FtsY and relocated to the vicinity of the C3 loop of SecY (Fig. 5). At that stage the affinity of the interaction of SRP and FtsY has decreased, which is also reflected in a slower association of the complex (Fig. 3d). This finding is at variance with a previous report where no effect of SecYEG on the SRP-FtsY interaction in the complex with an RNC was observed^38^. This discrepancy we attribute to the use of detergent to solubilize SecYEG^38^, whereas in our experiments we provided a phospholipid environment, which is essential for the SAS insertion and the interaction of FtsY with SecYEG^24^. In the post-transfer complex that we observed here, SRP and FtsY presumably are in the ‘closed/activated’ state^38,45^. According to recent structural and biochemical data, the heterodimer of the NG domains of Ffh and FtsY relocates to the distal end of the 4.5S RNA where GTPase activation and GTP hydrolysis take place^37,46–48^. Following GTP hydrolysis, SRP and FtsY are disassembled and recycled for another round of targeting.

We observed a sequential reduction of the affinity of SRP binding to RNCs and FtsY which is characteristic for the transition between pre- and post-transfer complexes (Fig. 6) and is distinct from the affinities reported for the binary complexes^1,2,18^. This suggests that in the post-transfer complex SRP may stay bound to FtsY in the complex with SecYEG, thus remaining in the vicinity of the membrane. This could be advantageous for targeting nascent proteins with consecutive transmembrane segments, as it may favor the interaction with SRP^4^. The formation of the pre- and post-transfer quaternary complexes provides an intermediate step in the targeting cycle which enables the ordered transfer of the nascent chain to the translocon and into the phospholipid bilayer.

## Material and Methods

### Material

The maleimide derivatives of Bodipy FL (Bpy), Alexa Fluor 488 or 555 (Alx), MDCC, and the non-fluorescent FRET acceptor QSY9, as well as Bodipy FL-NHS (Bpy) were purchased from Thermo Fisher Scientific. Total *E*. *coli* tRNA was from Roche Diagnostics. Radioactively labeled amino acids ([^14^C]Leu, [^3^H]Met) were from Perkin Elmer, Phusion polymerase from New England Biolabs, *E*. *coli* total lipids from Avanti polar lipids, and BioBeads SM-2 from BIO-RAD. Measurements were performed in buffer A (20 mM HEPES-KOH, pH 7.5, 70 mM NH_4_Cl, 30 mM KCl, 7 mM MgCl_2_, 10% (w/v) glycerol) at 25 °C, unless stated otherwise.

### Protein and RNA preparation and labeling

SRP protein Ffh was expressed from pET24 vector with 6xHis C-terminal purification tag in *E*. *coli* strain BL21(DE3)pLysS and purified by affinity chromatography^16^. 4.5S RNA was *in-vitro* transcribed by T7 RNA-polymerase from linearized DNA generated from plasmid pT7–4.5S RNA^14^. SRP was reconstituted from Ffh (10 µM) and 4.5S RNA (12 µM).

FtsY was expressed either from a pET9a vector with a C-terminal 6xHis-tag (for experiments with fluorescence-labeled FtsY) or a pET-SUMO vector with an N-terminal 6xHis-tag and a Ulp1 cleavage site just before the first Met of the protein coding sequence, in *E*. *coli* strain BL21(DE3)pLysS^16^. The N-terminal 6xHis-tag was cleaved by 6xHis-tagged Ulp1 SUMO protease (protease:protein 1:100) for 15 hours at 4 °C. Cleaved FtsY lacking the His-tag was isolated by nickel-affinity chromatography in the flow-through fraction.

SecYEG and membrane-scaffold protein MSP1D1 were expressed and purified as previously described^16^. SecYEG, SecY(S111C)EG and SecY(T179C)EG containing a N-terminally 6xHis-tagged SecE were expressed and purified according to established protocols^16^. To maintain SecYEG in soluble form all buffers were supplemented with 0.03% n-dodecyl-β-maltoside (DDM). MSP1D1 was expressed with an N-terminal 7xHis-tag in *E*. *coli* strain BL21(DE3) and isolated as described^21^.

Amino acid substitutions were generated by site-directed mutagenesis using Phusion polymerase, following the quick-change mutagenesis protocol (Agilent). Coupling of thiol-reactive fluorophores to engineered Cys residues was carried out according to the manufacturer’s protocol. Non-reacted maleimide was inactivated by incubation with 2-mercaptoethanol in ten-fold excess and removed by size-exclusion chromatography on Sephadex G-25. The labeling efficiency (usually 90–100%) was determined by absorbance measurements.

### Preparation of SecYEG-containing nanodiscs

Reconstitution of SecYEG in phospholipid-containing nanodiscs was performed as described^16^. The assembly reaction consisted of SecYEG, *E*. *coli* total lipids and membrane scaffold protein MSP1D1 in a ratio of 1:30:2. The amount of MSP1D1 and lipids was optimized to reduce the formation of empty nanodiscs and SecYEG aggregates. Incubation was for 1 hour on ice in buffer A without glycerol, supplemented with 0.1% DDM. Nanodisc assembly was initiated by the addition of BioBeads SM-2 and incubating the mixture overnight at 4 °C with gentle agitation. Subsequently, SecYEG-containing nanodiscs were isolated in a single homogeneous peak by size-exclusion chromatography on Superdex 200 PG 16/100 as described^21^.

The stoichiometry of SecYEG in the nanodiscs was determined by label-free quantitative mass spectrometry^49^. Purified nanodisc complexes were resolved by non-denaturing polyacrylamide electrophoresis, the bands were excised and processed by in-gel trypsin digest^50^. Tryptic fragments were analyzed in an LC-coupled ESI Q-ToF mass spectrometer under standard conditions. Raw data were processed by MaxQuant for peptide/protein identification. The ratio of SecY to MSP1D1 in our samples was 0.46 ± 0.06 (mean and SD were calculated from n = 8), indicating one copy of SecY per two copies of MSP1D1, i.e., a single copy of SecYEG per nanodisc.

### Preparation of ribosome-nascent-chain complexes

RNCs were prepared by translating truncated mRNAs in a reconstituted *in-vitro* translation system^1,2^. Ribosomes from *E*. *coli* MRE600, initiation factors (IF1, IF2 and IF3) and elongation factors (EF-Tu, EF-G) were purified according to established protocols^51^. Ribosomes labeled with MDCC at position S21C of mutant protein uL23 were prepared as described previously^2^, as were initiator tRNA f[^3^H]Met-tRNA^fMet^ and Bpy-[^3^H]Met-tRNA^fMet ^^52,53^. Total *E*. *coli* tRNA was aminoacylated with a mixture of 20 amino acids containing [^14^C]Leu^54^. mRNA was prepared by *in-vitro* transcription using T7 polymerase^55^.

Initiation complexes (IC) were prepared in 50 mM Tris-HCl, pH 7.5, 70 mM NH_4_Cl, 30 mM KCl, 7 mM MgCl_2_, 2 mM dithiothreitol (DTT), by incubating 70S ribosomes (1 µM) with initiator tRNA f[^3^H]Met-tRNA^fMet^ or Bpy-[^3^H]Met-tRNA^fMet^ (2 µM), a mixture of initiation factors IF1,IF2 and IF3 (1.5 µM each), mRNA (3 µM) and GTP (2 mM) for 1 h at 37 °C. Initiation efficiency (80–90%) was controlled by nitrocellulose filtration and ^3^H-radioactivity counting^53^. Ternary complex EF-Tu–GTP–aminoacyl-tRNA was prepared in the same buffer by incubating EF-Tu (84 µM), EF-Ts (0.04 µM), GTP (2 mM), phosphoenolpyruvate (6 mM), pyruvate kinase (0.2 mg/ml) and EF-G (2.5 µM) for 15 min at 37 °C. Then total *E*. *coli* aminoacyl-tRNA (42 µM; containing a four-fold excess of [^14^C]Leu-tRNA^Leu^ over Leu codons) was added, and ternary complex formation was completed by 2 min incubation at 37 °C. Translation was performed in HiFi buffer (50 mM Tris-HCl, pH 7.5, 70 mM NH_4_Cl, 30 mM KCl, 3.5 mM MgCl_2_, 0.5 mM spermidine, 8 mM putrescine, 2 mM DTT) by incubating IC (0.025 µM), EF-Tu–GTP–aminoacyl-tRNA (20 µM), EF-G (1.1 µM) and GTP (2 mM) for 5 min at 37 °C. RNCs were purified by centrifugation through a 1.1 M sucrose cushion in HiFi buffer for 2 h at 4 °C at 250,000 × g.

### Fluorescence measurements

Donor emission was monitored for the FRET pairs Bpy–Alx555 or Bpy–QSY9 by measuring emission spectra from 495 to 620 nm on excitation at 480 nm. Binding reactions were monitored by FRET between (Bpy)Lep-RNC and SRP(Alx)/SRP(QSY) or SecYEG(Alx) and contained RNC (10 nM), SRP (100 nM), FtsY (1 µM), SecYEG in nanodiscs (400 nM) and GDPNP (500 µM). Reactions monitoring FRET between SRP(Bpy) and SecYEG(Alx) contained RNC (20 nM), SRP (10 nM), FtsY (1 µM), SecYEG in nanodiscs (400 nM) and GDPNP (500 µM). To correct for donor fluorescence changes we performed donor-only measurements and corrected the FRET measurements by subtracting donor-only changes.

### Equilibrium titrations

Equilibrium titrations were performed in buffer A at 25 °C and monitored by donor fluorescence: Bpy fluorescence was measured at 520 nm on excitation at 480 nm; MDCC fluorescence was measured at 460 nm on excitation at 430 nm. The affinity of SRP binding to RNCs was determined by titrating (Bpy)Lep-RNC (4 nM) with SRP(Alx) in the presence of GDPNP (500 µM) in the presence of varying concentrations of SecYEG and a fixed concentration of FtsY (1 µM, if present). The binding of SRP to FtsY on the ribosome was monitored in the presence of either 70S ribosomes (500 nM) or Lep-RNC (10 nM) in combination with SecYEG in nanodiscs (300 nM) or empty nanodiscs (500 nM). SRP (25 nM), labeled with MDCC at position 235, was titrated with FtsY, labeled with acceptor fluorophore Bpy at position 487, and the change of MDCC donor fluorescence due to FRET was monitored.

For the evaluation of the equilibrium titrations we have applied a quadratic equation:

1 $$F(Y)=F0+\frac{({\rm{\Delta }}F)([{P}_{total}]+[X]+{K}_{d})-\sqrt{{([{P}_{total}]+[X]+{K}_{d})}^{2}-4[{P}_{total}][X]}}{2[{P}_{total}]}$$

where *F* is the donor fluorescence signal; *F0* is the initial fluorescence; *ΔF* is the fluorescence signal amplitude; *[P*_*total*_] is the total concentration of binding partner *A*; *[X]* is the concentration of added binding partner *B*; *K*_*d*_ is the equilibrium dissociation constant.

To analyze the type of interaction between SRP and SecYEG on the ribosome, we have performed further evaluation of the apparent affinities obtained by fluorescence titrations^7^:

2 $$app{K}_{d}(A)={K}_{d}(A)\frac{1+\frac{[B]}{{K}_{d}(B)}}{1+\frac{[B]}{\alpha {K}_{d}(B)}}$$

where *appK*_*d*_ is the apparent equilibrium dissociation constant of *A* (e.g. SRP) in the presence of *B* (e.g. SecYEG); *K*_*d*_*(A)* and *K*_*d*_*(B)* are the intrinsic equilibrium dissociation constants of the binding of *A* or *B*; [*B*] denotes the concentration of competitor; *α* is the modulation factor by which the *K*_*d*_*(A)* is changed in the presence of *B*^7,56^.

### Rapid kinetics

To monitor the association of FtsY and SRP in complex with vacant ribosomes or RNCs in the absence or presence of SecYEG (300 nM), 70S ribosomes (50 nM) or Lep-RNC (30 nM) in complex with SRP(MDCC) (500 nM) were rapidly mixed with FtsY(Bpy) (1 µM) in a stopped-flow apparatus. Bpy fluorescence was measured after passing a 530-nm cut-off filter.

### Proteinase digestion

SAS insertion into SecYEG was monitored by the protection of the N-terminal Bpy-Met against proteinase K cleavage, using an established protocol^16,21^. Lep50/75-RNCs labeled with Bpy at the N-terminal methionine of the nascent chain were incubated for 5 min at 37 °C in buffer A with SRP (2 µM), FtsY (4 µM), SecYEG or SecY39EG (2 µM) and GDPNP (500 µM) in different combinations followed by proteinase K (1.5 mg/ml) digestion for 30 min at 37 °C. Reactions were quenched by adding phenylmethylsulfonyl fluoride (45 mM), and nascent chains were released from peptidyl-tRNA by RNaseA digestion (10 mg/ml; 30 min at 37 °C). Samples (0.5 pmol) were analyzed by Tris-Tricine denaturing polyacrylamide gel electrophoresis^52^; protected peptides were visualized by Bpy fluorescence on FUJIFILM FLA-9000; contrast, saturation and brightness were adjusted with ImageJ.

## Electronic supplementary material

Supplementary Information
