# Supplementary material for: Cotranslational protein targeting to the membrane: Nascent-chain transfer in a quaternary complex formed at the translocon
Source: Sci Rep. 2018 Jul 2;8:9922. doi: 10.1038/s41598-018-28262-8 (PMC6028451; doi:10.1038/s41598-018-28262-8)
Supplement: Supplementary file 1 — Supplementary Information [file 41598_2018_28262_MOESM1_ESM.docx]

**Supplementary Information**

**Cotranslational protein targeting to the membrane: Nascent-chain transfer**

**in a quaternary complex formed at the translocon**

Albena Draycheva^#^, Sejeong Lee^#1^, Wolfgang Wintermeyer*

Max Planck Institute for Biophysical Chemistry, Department of Physical Biochemistry

37077Göttingen , Germany

* To whom correspondence should be addressed.

E-mail: [wolfgang.wintermeyer@mpibpc.mpg.de](mailto:wolfgang.wintermeyer@mpibpc.mpg.de)

^#^These authors contributed equally.

^1^ Present address: Chemistry Research Laboratory, University of Oxford,

OX1 3TA Oxford, UK

##
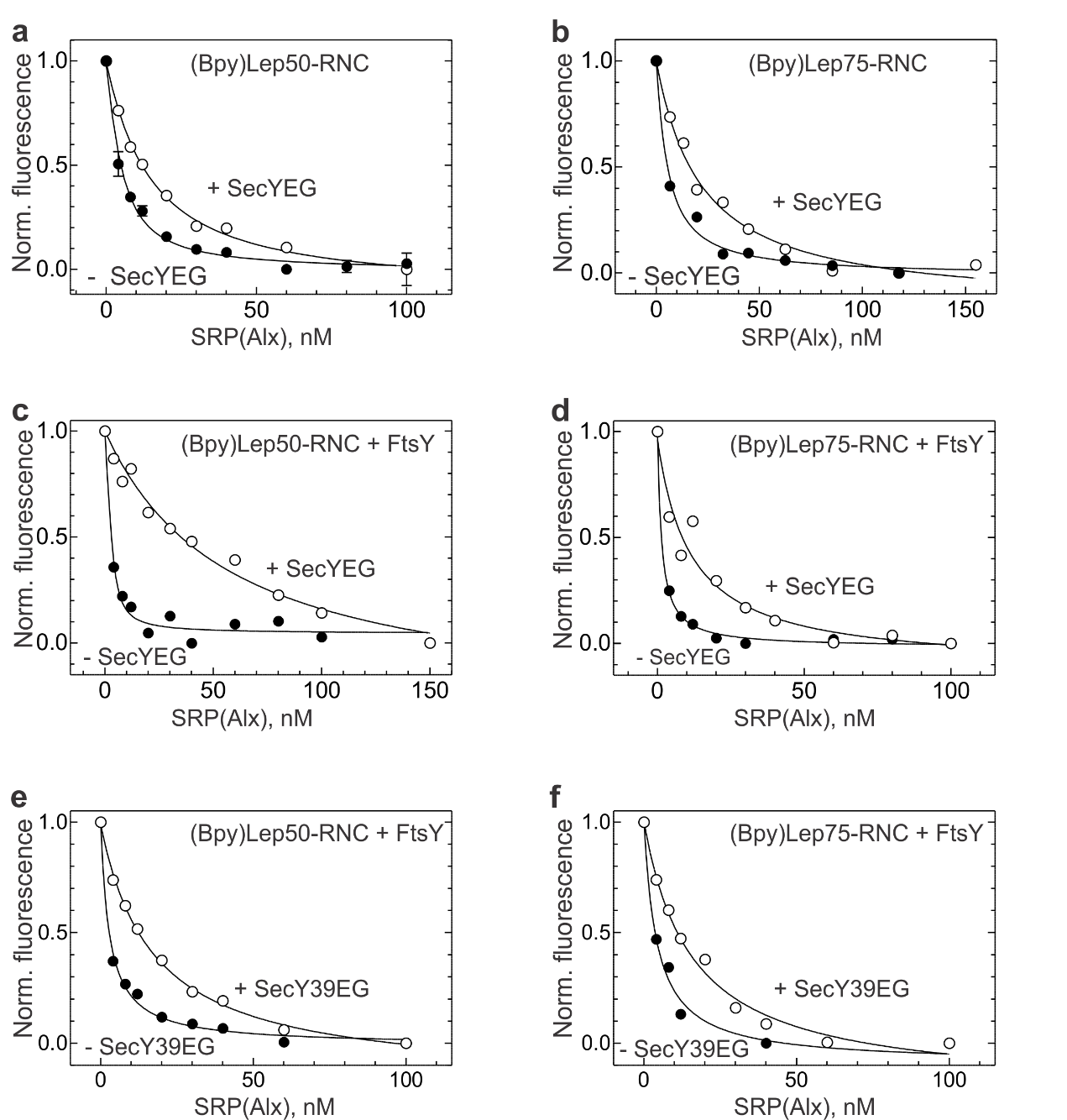


**Supplementary Figure S1.** Effect of SecYEG and FtsY on SRP binding to Lep-RNCs. Binding of SRP(Alx) to (Bpy)Lep50-RNC (4 nM) (**a**) or (Bpy)Lep75-RNC (4 nM) (**b**) was monitored by FRET in the absence (●) or presence (○) of SecYEG (up to 200 nM); titrations at intermediate concentrations of SecYEG (cf. Figure 1c) are not depicted for clarity. The fluorescence change is normalized to the initial and final signals. (**c, d, e, f**) Analogous SRP titrations of (Bpy)Lep50-RNC in the presence of SecYEG (**c**) or SecY39EG (**e**), or of (Bpy)Lep75-RNC in the presence of (up to 200 nM) SecYEG (**d**), or SecY39EG (**f**) were performed as in (a) and (b), but in the presence of FtsY (1 µM). Apparent K_d_ values were determined by fitting to equation (1) (Material and Methods). The quality of the fits varied from R^2^ = 0.88 (Lep50) to R^2^ = 0.57 (Lep75); the latter small R^2^ value is attributed to the small fluorescence change observed in the titrations with Lep75-RNC (cf. columns 2 and 3 in Fig. 1b).

**
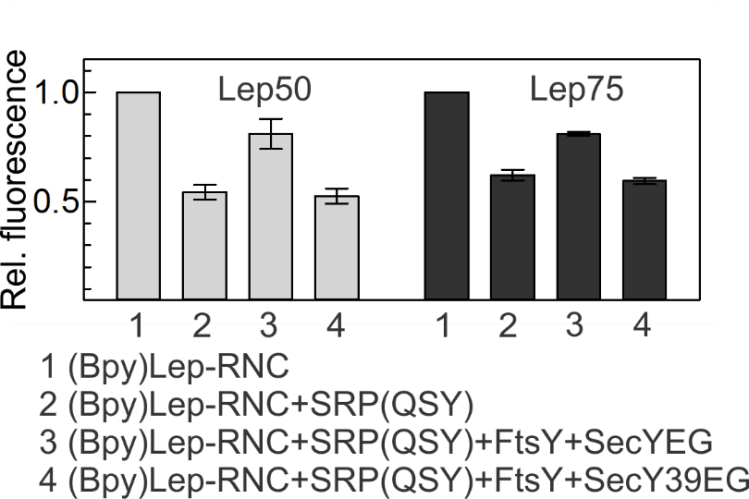
**

**Supplementary Figure S2.** SecYEG–FtsY induces a reorientation of SRP in the quaternary complex. The FRET change due to SRP binding to Lep50-RNC or Lep75-RNC is depicted. To (Bpy)Lep-RNC (10 nM) was added SRP(QSY) (100 nM); FtsY (1 µM) and SecYEG (400 nM); or FtsY and SecY39EG (400 nM). FRET between the Bpy donor at the N terminus of the nascent chain and the non-fluorescent acceptor QSY9 in the N domain of SRP protein Ffh (position 84). FRET was monitored by Bpy fluorescence and is normalized to the initial fluorescence measured prior to the addition of SRP(QSY9). Error margins represent SEM, n = 2–4.

**
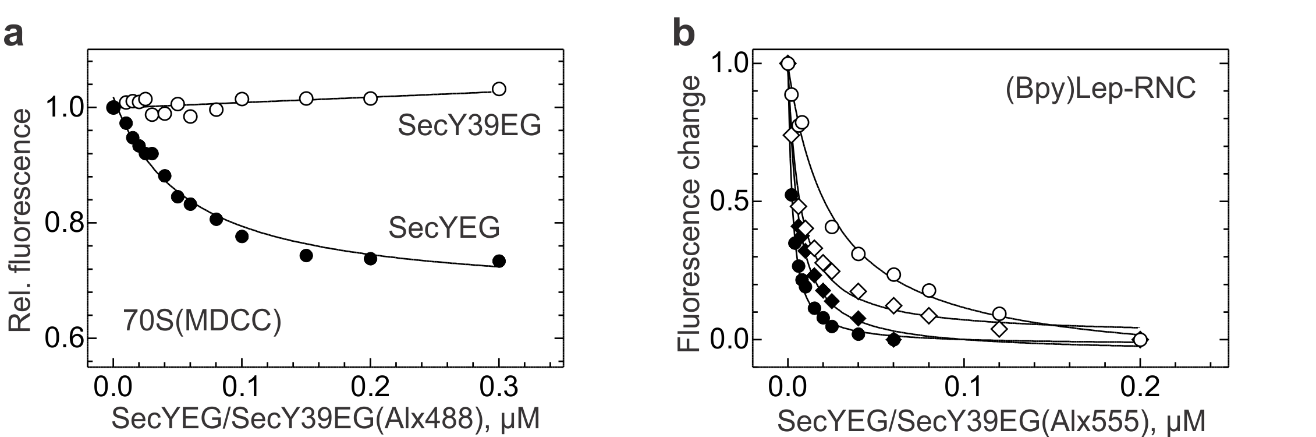
**

**Supplementary Figure S3.** Impaired ribosome binding of mutant SecY39EG. (**a**) Titration of vacant 70S ribosomes with SecYEG or mutant SecY39EG. Ribosomes labeled with FRET donor MDCC at position 21 of protein uL23 were titrated with SecYEG labeled with the FRET acceptor Alexa Fluor 488 (Alx488) at position 111 of SecY. Donor fluorescence was monitored and is plotted relative to the initial fluorescence. Fit of the data to equation (1) (Material and Methods) yielded K_d_ = 30 ± 20 nM for SecYEG. (**b**) Titration of Lep50-RNC and Lep75-RNC with SecYEG or SecY39EG. RNCs carrying nascent chains labeled with Bpy at the N terminus were titrated with SecYEG labeled with the FRET acceptor Alx555 at position 179 of SecY. The change of the donor fluorescence was normalized to the initial and final signal. The fit of the data to equation (1) (Material and Methods) yielded K_d_ = 2 ± 1 nM for SecYEG binding to Lep50-RNC (●); K_d_ = 25 ± 3 nM for SecY39EG binding to Lep50-RNC (○); K_d_ = 5 ± 1 nM for SecYEG binding to Lep75-RNC (◆); K_d_ = 7 ± 1 nM for SecY39EG binding to Lep75-RNC (◇). Error margins on K_d_ values represent errors of the fit.

**
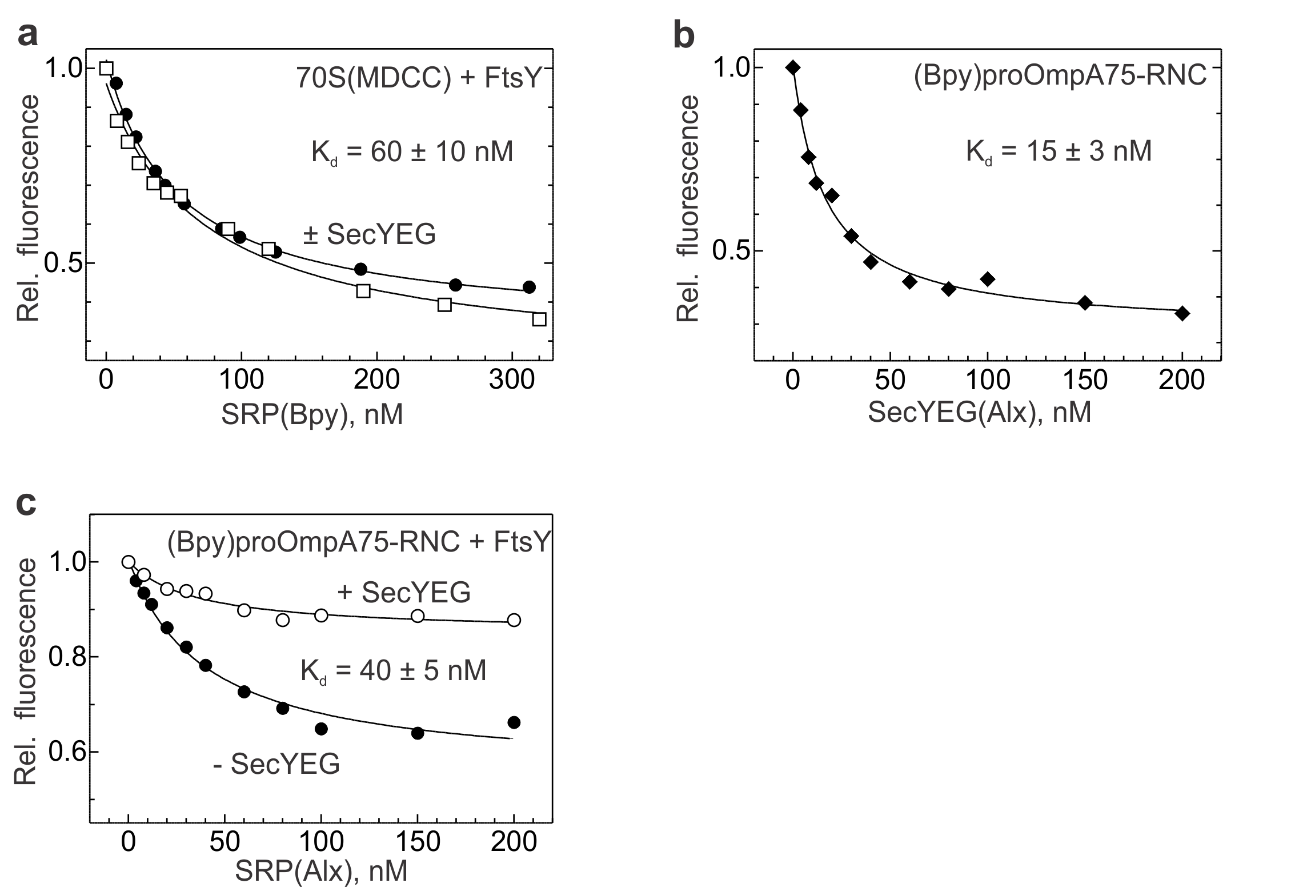
**

**Supplementary Figure S4.** Concurrent binding of SecYEG or SecYEG–FtsY and SRP to ribosomes or proOmpA-RNC. (**a**) Vacant 70S(MDCC) ribosomes (4 nM) were titrated with SRP(Bpy) in the presence of FtsY (1 µM) and in the absence (●) or presence of SecYEG (300 nM; □). K_d_ values were obtained by non-linear fitting to equation (1) (Material and Methods). (**b**) Titration of (Bpy)proOmpA75-RNC with SecYEG(Alx) labeled with Alexa555 on SecY monitoring FRET. (**c**) (Bpy)proOmpA75-RNC (4 nM) was titrated with acceptor-labeled SRP(Alx) in the presence of FtsY (1 µM) (●) or FtsY and SecYEG (200 nM; ○). The affinity of SRP binding to proOmpA75-RNC did not change upon the addition of SecYEG–FtsY. Error margins on K_d_ values represent errors of the fit.

**
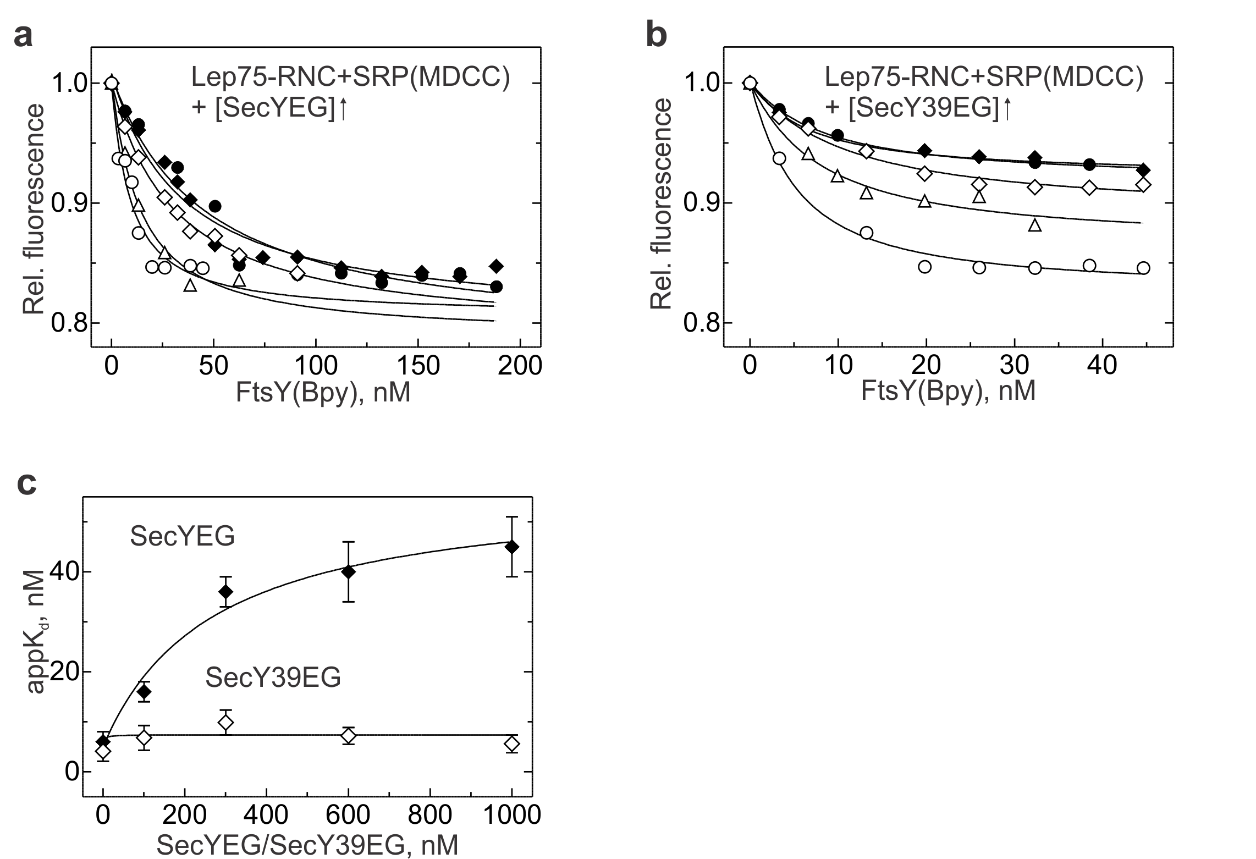
**

**Supplementary Figure S5.** SecYEG lowers the affinity of FtsY binding to SRP by competing for ribosome binding. Titrations of Lep75-RNC–SRP(MDCC) complex with FtsY(Bpy) in the presence of increasing concentrations of SecYEG (**a**) or SecY39EG (**b**). Concentrations of SecYEG or SecY39EG: 0 (○), 100 (Δ), 300 (◇), 600 (●) and 1000 nM (◆). Complex formation was monitored by FRET between the MDCC donor and the Bpy acceptor fluorophore. (**c**) Apparent K_d_ values were obtained by non-linear fitting of the titration curves in panels a and b to equation (2) (Material and Methods). Error margins represent errors of the fit.


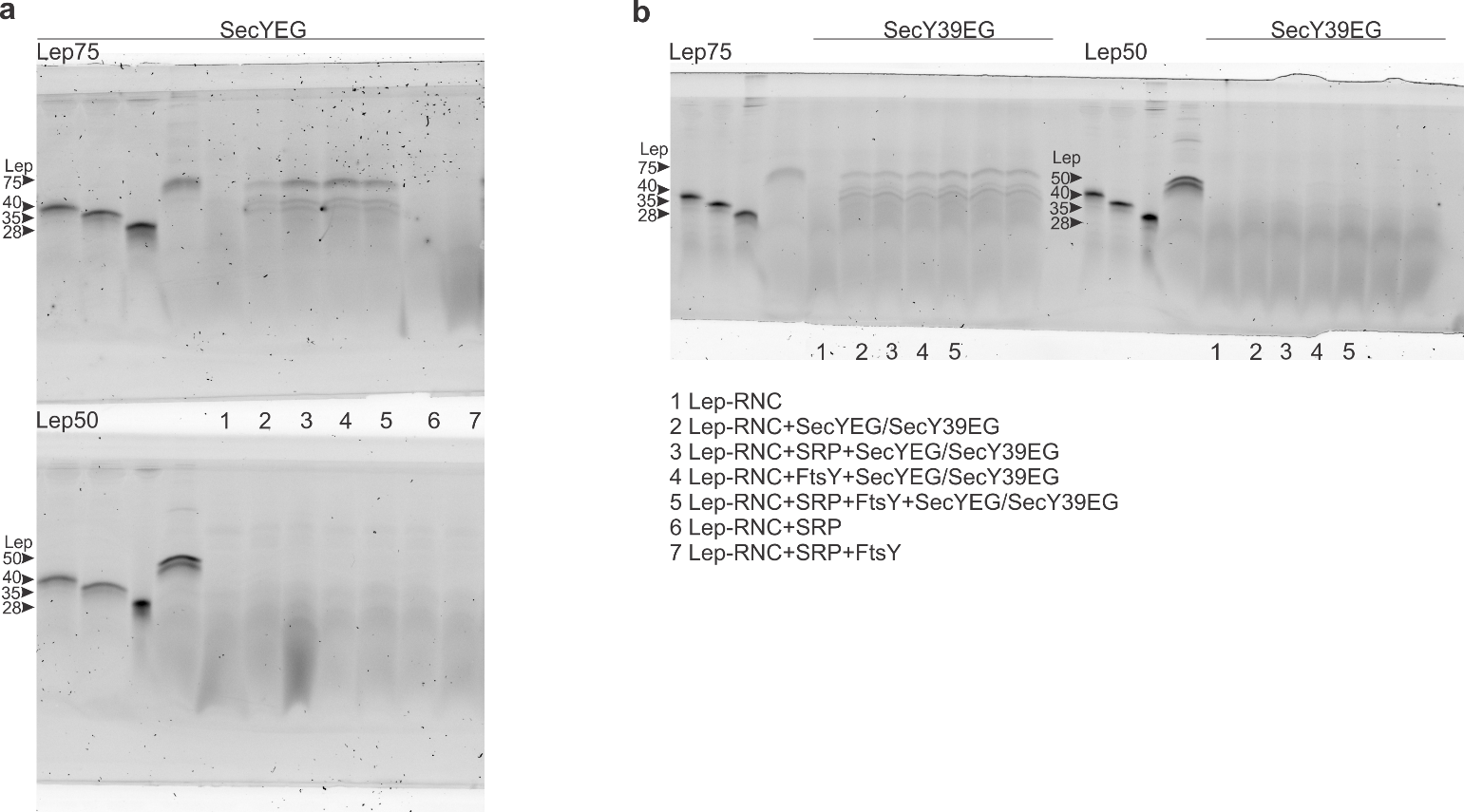


**Supplementary Figure S6.** Uncropped gel lanes of proteinase accessibility of Bpy-labeled Lep50 or Lep75 nascent chains in complexes with (**a**) SecYEG or (**b**) SecY39EG and SRP/FtsY. Lanes presented in Figure 4 are numbered 1 – 7 (a) and 1 – 5 (b).
